# Fourth Annual Report of the Board of Health, of Augusta, Ga.

**Published:** 1882-06-20

**Authors:** 


### *FOURTH ANNUAL REPORT OF THE BOARD OF HEALTH OF AUGUSTA, GA.*

For a copy of this able and interesting report we are indebted to Dr. Eugene Foster, the able and indefatigable President of the Board. "The report includes the annual address of the President of the Board; the annual report of the Secretary; the mortuary tables for the year 1881; the expenses of the Board for the past year; the estimate of expenses for the year 1882; details of the principal acts of this Board for 1881, with detailed reports of the Sanitary Inspectors."

